# Resting heart rate and antisocial behaviour: a Mendelian randomisation study

**DOI:** 10.1038/s41598-023-37123-y

**Published:** 2023-06-23

**Authors:** Lucy Karwatowska, Leonard Frach, Tabea Schoeler, Jorim J. Tielbeek, Joseph Murray, Eco de Geus, Essi Viding, Jean-Baptiste Pingault

**Affiliations:** 1 Great Ormond Street Institute of Child Health, University College London, 30 Guilford Street, London, WC1N 1EH UK; 2 Department of Clinical, Educational and Health Psychology, University College London, London, UK; 3 Department of Computational Biology, University of Lausanne, Lausanne, Switzerland; 4 Department of Complex Trait Genomics, VU University Amsterdam, Amsterdam, The Netherlands; 5 Postgraduate Program in Epidemiology, Federal University of Pelotas, Pelotas, Brazil; 6 Human Development and Violence Research Centre, Federal University of Pelotas, Pelotas, Brazil; 7 Department of Biological Psychology, Amsterdam Public Health Research Institute, Amsterdam, The Netherlands; 8 Developmental Risk & Resilience Unit, Division of Psychology & Language Sciences, University College London, London, UK; 9 Social, Genetic, and Developmental Psychiatry, King’s College London, De Crespigny Park, London, UK

**Keywords:** Human behaviour, Risk factors, Epidemiology

## Abstract

Observational studies frequently report phenotypic associations between low resting heart rate (RHR) and higher levels of antisocial behaviour (ASB), although it remains unclear whether this relationship reflects causality. To triangulate evidence, we conducted two-sample univariable Mendelian randomisation (MR), multivariable MR and linkage disequilibrium score regression (LDSC) analyses. Genetic data were accessed from published genome-wide association studies (GWAS) for RHR (n = 458,835) and ASB (n = 85,359) for the univariable analyses, along with a third GWAS for heart rate variability (HRV; n = 53,174) for all other analyses. Genome-wide significant (*p* < 5 × 10^−8^) single-nucleotide polymorphisms associated with RHR (n = 278) were selected as instrumental variables and the outcome was a composite measure of ASB. No causal association was observed between RHR and ASB (*B*_IVW_ =  − 0.0004, *p* = 0.841). The multivariable MR analyses including RHR and HRV also suggested no causal associations (*B*_IVW_ = 0.016, *p* = 0.914) and no genetic correlations between the heart rate measures and ASB were observed using LDSC (*r*_*g*_ = 0.057, *p* = 0.169). Sensitivity analyses suggested that our results are not likely to be affected by heterogeneity, pleiotropic effects, or reverse causation. These findings suggest that individual differences in autonomic nervous system functioning indexed by RHR are not likely to directly contribute to the development of ASB. Therefore, previously observed associations between RHR and ASB may arise from confounding, reverse causation, and/or additional study characteristics. Further causally informative longitudinal research is required to confirm our findings, and caution should be applied when using measures of RHR in interventions targeting ASB.

## Introduction

Antisocial behaviour (ASB), which includes aggression, rule-breaking and acts of violence, imposes a substantial economic and social burden on the individual, their community and wider society. Individuals who display high levels of ASB are at risk of lifelong adverse outcomes, such as poor mental health, substance misuse, criminal behaviour and unemployment^1–4^. Furthermore, up to half of individuals who display ASB in childhood continue to exhibit these behaviours through adolescence and adulthood^5–7^. Considering these long-term and pervasive adverse outcomes, it is important to understand the aetiology of ASB to inform early identification and evidence-based intervention efforts.

Numerous reviews exist on putative risk factors for ASB, which include environmental and neurobiological factors^8–11^. Physiological markers, such as those indexing autonomic nervous system (ANS) activity, are particularly important in elucidating potential mechanisms underlying the development of ASB^12,13^. Of these, resting heart rate (RHR), defined as the number of heart beats per minute while at rest, is the most well-studied. Observational studies frequently report a strong inverse relationship between RHR and ASB, where individuals with lower RHR display higher levels of various types of ASB, including child conduct problems, juvenile delinquency and adult violence^14–19^. Several meta-analyses have been conducted on this topic^20–23^, all reporting a robust association between the two traits, with one review stating that “resting heart rate is a possible causal risk factor for antisocial behavior”^20^.

Various potential mechanisms have been proposed to explain the relationship between RHR and ASB. The two main theories are the fearlessness^24^ and sensation-seeking hypotheses^25^. According to the fearlessness hypothesis, an individual with a low RHR has a higher threshold for experiencing fear than individuals with higher RHRs, partly due to attenuated ANS responses to aversive stimuli. The typical links between poor behavioural choices (e.g., aggression) and aversive stimuli (e.g., perceived punishment cues) are either not established or are insufficiently established in individuals with lower RHR. As such, an individual with lower RHR would have inappropriately low expectations of negative outcomes and be prone to repeat poor decision making. In support of this hypothesis, many behavioural experiments report that participants who show deficient fear conditioning and reduced anticipatory fear reactivity have lower RHRs and higher levels of ASB^26,27^.

The sensation-seeking hypothesis states that individuals with lower RHR have low basal ANS activity and are chronically hypo-aroused. Hypo-arousal is an unpleasant physiological state and therefore individuals with lower RHR seek to increase their arousal to a normal level by engaging in ASB. Sensation-seeking has been shown to be associated with both RHR and ASB, with some evidence suggesting that sensation-seeking is a mediator between these two factors^28–30^.

Although the link between RHR and ASB is well studied, questions remain over whether these two phenotypes are causally related. This is in part due to limitations in the existing literature which prevent the drawing of causal conclusions. A closer look at the studies included in the four meta-analyses^20–23^ shows that the majority of studies have used small and/or selective samples, which can produce unreliable and ungeneralisable estimates. In recent years, authors have attempted to include larger, unselected samples followed up over time. The findings from these studies are more inconsistent than those from earlier studies reporting a strong negative relationship between RHR and ASB, with some more recent studies confirming earlier findings^28,31–33^ and others suggesting no relationship between these two factors^12,34–37^. There is also a paucity of research adopting causal inference approaches to help overcome the inherent biases of these studies, including confounding and reverse causation. For example, only two studies have used genetically informed family-based methods and both found that the relationship between heart rate and ASB is entirely explained by genetic effects, i.e. genetic confounding^14,38^. Interestingly, these studies show evidence of genetic covariation between RHR and ASB, whereby children with a genetic liability for lower RHR also have a genetic liability for ASB.

Genetically informed methods can exploit the heritability of both RHR^39,40^ and ASB^41–43^. The two aforementioned genetically informed studies used methods which rely on knowledge of genetic relatedness between family members. Other genetically informed methods can be used to triangulate these findings by relying on different types of data and assumptions^44,45^. A useful genetically informed causal inference method is Mendelian randomisation (MR). MR is an instrumental variable approach that uses genetic variants (i.e. single nucleotide polymorphisms; SNPs) associated with an exposure of interest (e.g., SNPs associated with RHR) as instrumental variables to assess the effect of an exposure of interest (e.g., RHR) on an outcome (e.g. ASB). MR provides causal effect estimates under the classic instrumental variable assumptions: the genetic variants indexing the exposure must be (1) associated with the exposure (*relevance*); (2) independent of confounders of the exposure-outcome relationship (*exchangeability*); and (3) only associated with the outcome through the exposure (*exclusion restriction*)^46,47^. If these assumptions are met, a significant association between the genetic variants and the outcome suggests a causal relationship between the exposure and the outcome.

### Objectives

The current study will be the first to interrogate the potential causal effect of RHR on ASB using two-sample MR analyses. We will exploit powerful genetic data from two large, independent genome-wide association studies (GWAS) on RHR^48^ and ASB^49^ to provide a new type of evidence for triangulation with previous observational studies. The majority of evidence suggests an association between lower RHR and higher ASB, although research using more rigorous approaches has questioned the strength of this association. Given these findings, the current study aims to investigate whether RHR has a causal effect on ASB.

## Methods

### Study design

To identify potential causal effects of resting heart rate (RHR) on antisocial behaviour (ASB), we conducted two-sample Mendelian randomisation (MR) analyses using the inverse-variance weighted (IVW) estimator^47,50–55^. Univariable two-sample MR integrates summary level genetic data from two GWAS, one GWAS estimating the association between the single nucleotide polymorphism (SNPs) and the exposure (i.e., SNP-RHR), and the other, independent GWAS estimating the associations between the genetic variants and the outcome (i.e., SNP-ASB). The IVW estimator is a weighted regression of SNP-outcome effects on SNP-exposure effects where the intercept is constrained to zero and the weighting is based on the inverse of the variance, thereby reflecting the precision of each instrument^54^. The IVW estimator can be construed as a weighted average of the effect estimates across all SNPs (i.e., each SNP provides one estimate of the causal effect of interest).

We also ran a number sensitivity analyses to test for potential violations of the MR assumptions^46,47^. These included other MR methods such as MR Egger^56^, weighted median analysis^57^, MR Robust Adjusted Profile Score (RAPS)^58^, MR Pleiotropy RESidual Sum and Outlier (PRESSO)^59^ and contamination mixture methods^60^. We further checked for heterogeneity and horizontal pleiotropy using the MR Egger intercept, and we used the Steiger approach to rule out reverse causation^61^. A dictionary designed to provide a comprehensive and accessible overview of MR theory, methodology and interpretation is available online^62^.

The autonomic nervous system (ANS) controls both RHR and resting heart rate variability (HRV). Therefore, to assess alternative explanations, we conducted further analyses, including a multivariable MR analyses using data from a GWAS of HRV^63^. By adding a second indicator we better capture the individual differences in ANS functioning underlying low RHR, in particular high levels of cardiac vagal control. As such we were able to assess effects of RHR on ASB independent of cardiac vagal control, for instance due to cardiac sympathetic control, and therefore elucidate possible mechanisms of ANS activity. As a positive control we also conducted MR with HRV as an alternative outcome, assuming a negative causal effect between RHR and HRV. Finally we conducted linkage disequilibrium (LD) score regression analyses^64,65^ to estimate genetic correlations between the heart rate measures and ASB. We followed the Strengthening the Reporting of Observational Studies in Epidemiology—Mendelian Randomization (STROBE-MR) guidelines^66^ (see Supplementary Table S1).

### Data sources and measures

The current study used data from three summary statistics files. There was no sample overlap between the RHR and the other two GWAS and limited overlap (potential n = 1300) between the HRV and ASB GWAS. Further information on the cohorts and measures used in all the original GWAS is available in Supplementary Table S2. Ethical approval for each GWAS was obtained by the authors of the original studies^48,49,63^.

#### Exposure measures

##### Resting heart rate

The summary statistics for RHR were obtained from the largest and most recent GWAS on RHR^48^, which included 458,835 individuals from the UK Biobank^67^. The original GWAS controlled for smoking but as smoking is an important covariate for RHR and ASB^20,30^ and was not controlled for in the ASB GWAS we asked the study authors to rerun the analysis without controlling for smoking. RHR is expressed in beats per minute. Further information is available in the Supplementary Materials.

##### Resting heart rate variability

Resting HRV captures the vagal effects on the sinoatrial node co-determining RHR and has also been considered a potential marker of ASB^69,70^. We obtained summary statistics for HRV from the Genetic Variance in Heart Rate Variability (VgHRV) Consortium GWAS^63^ of 53,174 participants (see Supplementary Materials).

#### Outcome measures

##### Antisocial behaviour

Summary statistics for ASB were obtained from the Broad Antisocial Behaviour Consortium (BroadABC) GWAS, which includes 85,359 individuals^49^. The ASB measures from these samples covered a broad range of behaviours including conduct disorder, aggression, and delinquency using study-specific scales in different age groups (see Supplementary Table S2 for further information).

### SNP selection

Prior to the main analyses, we conducted quality control procedures on the GWAS summary statistics, including harmonisation and clumping using default parameters from the R package *TwoSampleMR*^71^ (see Supplementary Materials). The same clumping and harmonisation parameters were used in all univariable and multivariable MR analyses.

### Statistical analyses

We performed all MR analyses in *R (version 4.2.3*^72^) using the *TwoSampleMR (version 0.5.7*^71^) and *MendelianRandomization (version 0.7.0*^73^) packages. The LD score regression analyses were conducted using a publicly available command line tool available on GitHub: LDSC (https://github.com/bulik/ldsc)^64,65^.

## Results

### Univariable MR analyses between resting heart rate and antisocial behaviour

After harmonisation, 300 genetic variants were available in both exposure and outcome datasets, of which eight non-inferable palindromic SNPs with intermediate allele frequencies were removed. For the remaining 292 variants, we investigated the direction of their effects using Steiger filtering. Fourteen SNPs showed higher associations with the outcome than the exposure and were removed, leaving 278 variants. The MR analyses of RHR on ASB using the IVW method did not support a causal effect, with an estimate close to zero and the 95% confidence intervals including the null (N_SNPs_ = 278; *B*_*IVW*_ =  − 0.0004; 95% CI − 0.004, 0.004; *p* = 0.841; see Table 1).

**Table 1:** Results from the univariable Mendelian randomisation analyses on resting heart rate and antisocial behaviour.

Method	*N*_*SNPs*_	*B*	*SE*	*p*	95% CIs	
					Lower	Upper
IVW	278	− 0.0004	0.002	0.841	− 0.004	0.004
MR Egger	278	− 0.0007	0.004	0.849	− 0.008	0.007
Weighted median	278	− 0.0039	0.003	0.199	− 0.010	0.002
MR RAPS	278	− 0.0009	0.002	0.617	− 0.005	− 0.003
MR PRESSO	278	− 0.0004	0.002	0.833	− 0.004	0.003
Contamination mixture	278	− 0.0023	0.003	0.452	− 0.008	0.004

### Sensitivity analyses

Table 1 also summarises the results obtained using other estimators. The IVW and MR Egger *Q* statistics revealed significant heterogeneity in the estimates (*Q* = 339.32, *p* = 0.024 and *Q* = 339.32, *p* = 0.027 respectively). No directional pleiotropic effects were observed from the MR Egger intercept (intercept = 0.0000185; *p* = 0.985) or MR PRESSO global test for pleiotropy (RSS_obs_ = 252.642, p = 0.872), which supports the exclusion restriction assumption.

The MR Steiger test revealed that associations between the genetic instruments and the exposure were 7.49 times higher than with the outcome (*R*^2^_*EXP*_ = 0.05, *R*^2^_*OUT*_ = 0.007), and therefore we were able to assume that if a causal effect existed it was from the exposure to the outcome, instead of the outcome to the exposure.

The results did not change when conducting leave-one-out analyses using IVW (*p*_*min*_ = 0.605) and MR Egger (*p*_*min*_ = 0.598). The genetic instruments had a high average *F* statistic of 84.20 (range = 27.30–1185.15) and the *I*^*2*^_*GX*_ statistic of 0.99 further showed that the results were not affected by weak instrument bias^57^.

### Univariable MR analysis between heart rate variability and antisocial behaviour

To investigate alternative explanations for the association reported by previous research between RHR and ASB, we conducted additional analyses including univariable MR with HRV, multivariable MR and LD score regression. The results from the univariable MR analyses suggested no causal effect of any of the three measures of HRV on ASB (Supplementary Table S3).

### Multivariable MR analysis with resting heart rate and heart rate variability

We also performed multivariable MR using both RHR and HRV (root mean square of the successive differences of inter beat intervals; RMSSD) as exposures. Variants that were significant in either of the two exposure datasets and were available in *both* datasets were retrieved and we performed clumping and harmonisation, resulting in a final set of 18 SNPs. The results from the multivariable MR did not support a causal effect of RHR on ASB when cardiac vagal effects were accounted for (Supplementary Table S4).

### Univariable MR analysis between resting heart rate and heart rate variablility

As a positive control, we conducted a MR analysis using RHR as the exposure and an alternative outcome that we assumed would be causally related to RHR, resting HRV. The results from these results were significant (N_SNPs_ = 206; *B*_*IVW*_ =  − 0.014; 95% CI − 0.017, − 0.011; *p* < 0.001; see Supplementary Table S5).

### LD score regression

Finally, we performed LD score regression to calculate genetic correlations between the heart rate measures and ASB, using default parameters (see Supplementary Materials). There were significant genetic correlations between RHR and HRV but we did not find any significant genetic correlations between either heart rate measure and ASB (Supplementary Tables S6, S7).

## Discussion

In these preliminary analyses using two-sample Mendelian randomisation (MR), we report no significant effects for resting heart rate (RHR) on antisocial behaviour (ASB). Sensitivity analyses suggested that these results are unlikely to be affected by heterogeneity, pleiotropy and/or weak instrument bias. Additional analyses using a measure that captures the vagal contribution to RHR, heart rate variability (HRV), also did not produce any significant effects and there were no significant genetic correlations between any measure of heart rate and ASB.

In line with two prior studies that controlled for unmeasured confounders^14,38^, using a twin and a co-relative control design, our results do not support the hypothesis that the often observed association between RHR and ASB is directly causal. The null findings in the current study and the discrepancy between these and the phenotypic associations reported in previous research lend themselves to several alternative explanations.

First, it may be that the relationship between RHR and ASB *is* causal but the current study was not able to detect this either on account of limited power due to the sample size of the outcome GWAS (n = 85,359) or because RHR has a causal effect on specific, potentially “more extreme”, forms of ASB than those included in this study. The most recent meta-analysis on RHR and ASB found significant evidence of heterogeneity of effects, with the effect of RHR on ASB being largest for the most violence offenders and those with psychopathy^21^. Although the phenotype used here included “more extreme” forms of ASB (e.g., violent and sexual crimes) and clinical samples, these measures were combined with other “less extreme” forms (e.g., delinquency) to create a broad measure of ASB. Therefore, it is possible that we were not able to detect a potentially true causal effect on specific forms of ASB. It should be noted that the only three other genetically informed studies found no evidence of an effect of RHR on ASB in childhood^14^ or in adulthood^38^ nor evidence of a genetic correlation between RHR and childhood aggression^74^. Future research should aim to investigate heterogeneity in the relationship between RHR and ASB by considering specific phenotypes of ASB.

Another potential explanation is that the relationship between RHR and ASB *is not* causal but may arise in part due to issues in data quality and/or publication bias in previous research. In terms of data quality, many existing studies have included small and/or non-representative samples. In the most recent meta-analysis on RHR and ASB^21^, over half of the studies included data from fewer than 100 participants (61%; n = 62). The funnel plots also showed evidence of publication bias, whereby extreme negative findings were more likely to have been identified and included in the meta-analysis than studies that reported either a null or positive effect of RHR on ASB. The availability of larger and more representative datasets, such as those included in the current analyses, lends itself to future research overcoming these data quality issues.

A third explanation is that the association reported in previous research is driven by genetic confounding. Indeed, previous genetically informed studies suggest that the association between RHR and ASB is entirely explained by genetic effects^14,38^. However, using LDSC, we were unable to support this hypothesis. Our results suggested no evidence of genetic correlation between any measure of heart rate and ASB, in line with a recent GWAS on childhood aggression which also found no genetic correlation with RHR^74^. It should be noted that LDSC relies on common SNPs which only capture a fraction of the heritability of RHR and ASB so we cannot exclude that future studies using larger samples and/or including rarer variants may detect significant genetic correlations between RHR and ASB.

A fourth explanation for the association between RHR and ASB is that it is driven by other confounders which are not adequately accounted for in previous research. In another recent meta-analysis^20^ over three quarters of the effect sizes included adjusted for no confounders (77%; n = 89). It may be, for example, that sensation-seeking behaviour, which has been found to be associated with both RHR and ASB^28–30^, could be a “common cause” for both low RHR and high ASB. These alternative causes of the association between RHR and ASB need to be investigated fully as simply adjusting for a larger number of putative confounders poses the risk of conditioning on mediators and colliders.

Potential time-varying confounders have also not often been considered previously. Repeated measures enable the examination of the temporal ordering of variables. By using causal inference methods it is possible to control for time-fixed unmeasured confounding (e.g. fixed effects analyses) and/or measured time-varying confounders (e.g. g-methods). However, there is currently a lack of longitudinal studies looking at RHR and ASB. In the same meta-analysis, nine in ten of the studies included were cross-sectional (90%; n = 91). Indeed, we are aware of only eight studies with moderate sample sizes (i.e. including more than 100 participants) that use longitudinal data^14,28,32,33,38,75–77^. Five of these studies found an inverse association between RHR and ASB^14,28,32,33,76^ and three studies found no relationship^38,75,77^. These results highlight the inconsistencies in the literature which may arise in part due to differences in study design, RHR and ASB measurement, analyses and confounder adjustment. Of note, only two of these studies employed causal inference methods^14,38^, with both using family-based genetically informed methods. In order to draw causal conclusions, triangulation using methods that utilise different but complementary assumptions is needed^44,45^. The current study adds to the existing evidence by relying on instrumental variable assumptions. However, further analyses with large, longitudinal datasets using causal inference methods is required to disentangle this relationship further.

Despite limited confounder adjustment, the use of small sample sizes, cross-sectional data, and evidence of publication bias, it has been argued that RHR measures could be incorporated into risk assessments and interventions for ASB^20,21^. The null findings from the current study and the lack of high-quality evidence from previous research suggest that, although RHR may still be a robust indicator of ASB, RHR should not be interpreted as a causal risk factor for ASB until more rigorous, longitudinal research is conducted.

### Strengths and limitations

The current study has some key strengths, such as utilising data from two, large GWAS and using MR analyses, which can help strengthen causal inference when instrumental variable assumptions are met. However, we must consider certain limitations. As mentioned above, the ASB GWAS had a relatively small sample size (n = 85,359) in comparison to other GWAS and reported a SNP heritability of 8.4%^49^. Therefore, the current study may not have been powered to detect small causal effects. However, the clinical utility of such small effects, for instance using RHR as a basis to diagnose or intervene on ASB, is uncertain. As is often the case, once more data are available the analyses should be updated using GWAS with larger sample sizes.

Another potential limitation that has already been discussed is that the phenotypic measurements in both the exposure and the outcome GWAS were heterogeneous. The exposure GWAS used a measure of RHR which was averaged over multiple measures and the outcome GWAS combined questionnaires that captured a broad range of ASB types. Therefore, we may not have been able to detect an association between RHR and ASB due to the heterogeneity in the measurements used.

Furthermore, it should be noted that the exposure GWAS used an older sample than the outcome GWAS sample, meaning that the SNP-exposure associations were measured later than the SNP-outcome associations. MR estimates are often interpreted as lifetime exposures but the effect of this on MR results is an issue of ongoing debate^78,79^. However, it may be that the genetic effects of RHR on ASB are different over the life course (e.g. during adolescence) and we were not able to detect this in the current study.

Finally, although more of a concern for significant MR findings, some of the instrumental variable assumptions of MR are not verifiable. To be confident that the assumptions were supported, we used genetic variants that reached genome-wide significance; checked for high *F* statistics to support the relevance assumption; ensured the absence of significant horizontal pleiotropy to support the exclusion restriction assumption; and used a positive control.

## Conclusions

We found no significant genetic correlation nor a causal link between RHR and ASB in preliminary analyses using currently available summary statistics. Therefore, our results do not support that the often-reported association between RHR and ASB is causal. We suggest that the association reported by observational studies may be due to biased estimates resulting from small, selective samples, publication bias, and from inadequate control of genetic and environmental confounders. Future research should aim to use larger samples and appropriately control for potential confounders by using longitudinal data and more robust study designs (e.g., discordant monozygotic twin studies) and statistical analyses (e.g., within-person fixed effects, g-methods). Only by adopting a range of causal inference methods will researchers be able to further understand whether there is a causal relationship between RHR and ASB.

## Supplementary Information

Supplementary Information.

## Data Availability

The secondary data used in the current study are available either from the public GWAS catalog (HRV: https://www.ebi.ac.uk/gwas/publications/28613276) or directly from the study authors (RHR: Dr Zhaozhong Zhu; zhz586@mail.harvard.edu; ASB: Dr Jorim J. Tielbeek; j.tielbeek@amsterdamumc.nl). The scripts used to clean the data and produce the results will be available on GitHub (https://github.com/lk1373190/asb_mr).

## References

[1] Bevilacqua L, Hale D, Barker ED, Viner R (2017). Conduct problems trajectories and psychosocial outcomes: A systematic review and meta-analysis. Eur. Child Adolesc. Psychiatry..

[2] Colman I (2009). Outcomes of conduct problems in adolescence: 40 year follow-up of national cohort. BMJ.

[3] Huesmann LR, Dubow EF, Boxer P (2009). Continuity of aggression from childhood to early adulthood as a predictor of life outcomes: Implications for the adolescent-limited and life-course-persistent models. Aggress. Behav..

[4] Piquero AR, Daigle LE, Gibson C, Piquero NL, Tibbetts SG (2007). Are life-course-persistent offenders at risk for adverse health outcomes?. J. Res. Crime Delinq..

[5] Barker ED, Maughan B (2009). Differentiating early-onset persistent versus childhood-limited conduct problem youth. Am. J. Psychiatry.

[6] Maughan B, Kim-Cohen J (2005). Continuities between childhood and adult life. Br. J. Psychiatry.

[7] Moffitt TE (1993). Adolescence-limited and life-course-persistent antisocial behavior: A developmental taxonomy. Psychol. Rev..

[8] Derzon JH (2010). The correspondence of family features with problem, aggressive, criminal, and violent behavior: A meta-analysis. J. Exp. Criminol..

[9] Hawkins DJ (2000). Predictors of Youth Violence.

[10] Murray J, Farrington DP (2010). Risk factors for conduct disorder and delinquency: Key findings from longitudinal studies. Can. J. Psychiatry.

[11] Jaffee SR, Strait LB, Odgers CL (2012). From correlates to causes: Can quasi-experimental studies and statistical innovations bring us closer to identifying the causes of antisocial behavior?. Psychol. Bull..

[12] Fanti KA (2018). Understanding heterogeneity in conduct disorder: A review of psychophysiological studies. Neurosci. Biobehav. Rev..

[13] Matthys W, Vanderschuren LJMJ, Schutter DJLG (2013). The neurobiology of oppositional defiant disorder and conduct disorder: Altered functioning in three mental domains. Dev. Psychopathol..

[14] Baker LA (2009). Resting heart rate and the development of antisocial behavior from age 9 to 14: Genetic and environmental influences. Dev. Psychopathol..

[15] Bergstrøm H, Farrington DP (2018). “The beat of my heart”: The relationship between resting heart rate and psychopathy in a prospective longitudinal study. J. Crim. Psychol..

[16] Jennings JR, Pardini DA, Matthews KA (2017). Heart rate, health, and hurtful behavior. Psychophysiology.

[17] Raine A, Venables PH, Mednick SA (1997). Low resting heart rate at age 3 years predisposes to aggression at age 11 years: Evidence from the mauritius child health project. J. Am. Acad. Child Adolesc. Psychiatry.

[18] Schoorl J, Van Rijn S, De Wied M, Van Goozen SHM, Swaab H (2016). Variability in emotional/behavioral problems in boys with oppositional defiant disorder or conduct disorder: The role of arousal. Eur. Child Adolesc. Psychiatry.

[19] Wadsworth MEJ (1976). Delinquency, pulse rates and early emotional deprivation. Br. J. Criminol..

[20] Portnoy J, Farrington DP (2015). Resting heart rate and antisocial behavior: An updated systematic review and meta-analysis. Aggress. Violent. Behav..

[21] de Looff PC (2022). Heart rate and skin conductance associations with physical aggression, psychopathy, antisocial personality disorder and conduct disorder: An updated meta-analysis. Neurosci. Biobehav. Rev..

[22] Lorber MF (2004). Psychophysiology of aggression, psychopathy, and conduct problems: A meta-analysis. Psychol. Bull..

[23] Ortiz J, Raine A (2004). Heart rate level and antisocial behavior in children and adolescents: A meta-analysis. J. Am. Acad. Child Adolesc. Psychiatry.

[24] Raine A, Raine A (1993). Crime and the nature of psychopathology. The Psychopathology of Crime.

[25] Raine A (2002). Biosocial studies of antisocial and violent behavior in children and adults: A review. J. Abnorm. Child Psychol..

[26] Gao Y, Raine A, Venables PH, Dawson ME, Mednick SA (2010). Association of poor childhood fear conditioning and adult crime. Am. J. Psychiatry.

[27] López R, Poy R, Patrick CJ, Moltó J (2013). Deficient fear conditioning and self-reported psychopathy: The role of fearless dominance. Psychophysiology.

[28] Hammerton G (2018). Low resting heart rate, sensation seeking and the course of antisocial behaviour across adolescence and young adulthood. Psychol. Med..

[29] Portnoy J (2014). Heart rate and antisocial behaviour: The mediating role of impulsive sensation seeking. Criminology.

[30] Sijtsema JJ (2010). Mediation of sensation seeking and behavioral inhibition on the relationship between heart rate and antisocial behavior: The TRAILS study. J. Am. Acad. Child Adolesc. Psychiatry.

[31] Armstrong T (2019). Skin conductance, heart rate and aggressive behavior type. Biol. Psychol..

[32] Latvala A, Kuja-Halkola R, Almqvist C, Larsson H, Lichtenstein P (2015). A Longitudinal study of resting heart rate and violent criminality in more than 700,000 men. JAMA Psychiatry.

[33] Murray J (2016). Low resting heart rate is associated with violence in late adolescence: A prospective birth cohort study in Brazil. Int. J. Epidemiol..

[34] Fanti KA (2019). Psychophysiological activity and reactivity in children and adolescents with conduct problems: A systematic review and meta-analysis. Neurosci. Biobehav. Rev..

[35] Oldenhof H (2019). Baseline autonomic nervous system activity in female children and adolescents with conduct disorder: Psychophysiological findings from the FemNAT-CD study. J. Crim. Just..

[36] Prätzlich M (2019). Resting autonomic nervous system activity is unrelated to antisocial behaviour dimensions in adolescents: Cross-sectional findings from a European multi-centre study. J. Crim. Just..

[37] Kavish N, Fu QJ, Vaughn MG, Qian Z, Boutwell BB (2019). Resting heart rate and psychopathy revisited: Findings from the add health survey. Int. J. Offender Ther. Comp. Criminol..

[38] Kendler KS, Lönn SL, Sundquist J, Sundquist K (2021). The causal nature of the association between resting pulse in late adolescence and risk for internalizing and externalizing disorders: A co-relative analysis in a national male Swedish sample. Psychol. Med..

[39] De Geus EJC, Kupper N, Boomsma DI, Snieder H (2007). Bivariate genetic modeling of cardiovascular stress reactivity: Does stress uncover genetic variance?. Psychosom. Med..

[40] Eppinga RN (2016). Identification of genomic loci associated with resting heart rate and shared genetic predictors with all-cause mortality. Nat. Genet..

[41] Lewis GJ, Plomin R (2015). Heritable influences on behavioural problems from early childhood to mid-adolescence: Evidence for genetic stability and innovation. Psychol. Med..

[42] Polderman TJC (2015). Meta-analysis of the heritability of human traits based on fifty years of twin studies. Nat. Genet..

[43] Salvatore JE, Dick DM (2018). Genetic influences on conduct disorder. Neurosci. Biobehav. Rev..

[44] Munafò MR, Smith GD (2018). Robust research needs many lines of evidence. Nature.

[45] Lawlor DA, Tilling K, Davey Smith G (2017). Triangulation in aetiological epidemiology. Int. J. Epidemiol..

[46] Burgess S, Small DS, Thompson SG (2017). A review of instrumental variable estimators for Mendelian randomization. Stat. Methods Med. Res..

[47] Davey Smith G, Hemani G (2014). Mendelian randomization: Genetic anchors for causal inference in epidemiological studies. Hum. Mol. Genet..

[48] Zhu Z (2019). Genetic overlap of chronic obstructive pulmonary disease and cardiovascular disease-related traits: A large-scale genome-wide cross-trait analysis. Respir. Res..

[49] Tielbeek JJ (2022). Uncovering the genetic architecture of broad antisocial behavior through a genome-wide association study meta-analysis. Mol. Psychiatry..

[50] Burgess S, Scott RA, Timpson NJ, Davey Smith G, Thompson SG (2015). Using published data in Mendelian randomization: A blueprint for efficient identification of causal risk factors. Eur. J. Epidemiol..

[51] Burgess S, Timpson NJ, Ebrahim S, Davey Smith G (2015). Mendelian randomization: Where are we now and where are we going?. Int. J. Epidemiol..

[52] Pierce BL, Burgess S (2013). Efficient design for mendelian randomization studies: Subsample and 2-sample instrumental variable estimators. Am. J. Epidemiol..

[53] Smith GD, Ebrahim S (2003). ‘Mendelian randomization’: Can genetic epidemiology contribute to understanding environmental determinants of disease?. Int. J. Epidemiol..

[54] Burgess S, Butterworth A, Thompson SG (2013). Mendelian randomization analysis with multiple genetic variants using summarized data. Genet. Epidemiol..

[55] Burgess S (2020). Guidelines for performing Mendelian randomization investigations. Wellcome Open Res..

[56] Bowden J, Smith GD, Burgess S (2015). Mendelian randomization with invalid instruments: Effect estimation and bias detection through Egger regression. Int. J. Epidemiol..

[57] Bowden J, Davey Smith G, Haycock PC, Burgess S (2016). Consistent estimation in Mendelian randomization with some invalid instruments using a weighted median estimator. Genet. Epidemiol..

[58] Zhao Q, Wang J, Hemani G, Bowden J, Small DS (2020). Statistical inference in two-sample summary-data Mendelian randomization using robust adjusted profile score. Ann. Stat..

[59] Verbanck M, Chen C-Y, Neale B, Do R (2018). Detection of widespread horizontal pleiotropy in causal relationships inferred from Mendelian randomization between complex traits and diseases. Nat. Genet..

[60] Burgess S, Foley CN, Allara E, Staley JR, Howson JMM (2020). A robust and efficient method for Mendelian randomization with hundreds of genetic variants. Nat. Commun..

[61] Hemani G, Tilling K, Smith GD (2017). Orienting the causal relationship between imprecisely measured traits using GWAS summary data. PLoS Genet..

[62] *Mendelian Randomization Dictionary*. https://mr-dictionary.mrcieu.ac.uk/.

[63] Nolte IM (2017). Genetic loci associated with heart rate variability and their effects on cardiac disease risk. Nat. Commun..

[64] Bulik-Sullivan B (2015). An atlas of genetic correlations across human diseases and traits. Nat. Genet..

[65] Bulik-Sullivan B (2015). LD Score regression distinguishes confounding from polygenicity in genome-wide association studies. Nat. Genet..

[66] Skrivankova VW (2021). Strengthening the reporting of observational studies in epidemiology using mendelian randomisation (STROBE-MR): Explanation and elaboration. BMJ..

[67] Allen NE, Sudlow C, Peakman T, Collins R (2014). UK Biobank data: Come and get it. Sci. Transl. Med..

[69] Beauchaine TP, Thayer JF (2015). Heart rate variability as a transdiagnostic biomarker of psychopathology. Int. J. Psychophysiol..

[70] Beauchaine TP (2019). Respiratory sinus arrhythmia reactivity across empirically based structural dimensions of psychopathology: A meta-analysis. Psychophysiology.

[71] Hemani G (2018). The MR-base platform supports systematic causal inference across the human phenome. eLife.

[72] R Core Team. *R: A Language and Environment for Statistical Computing* (2019).

[73] Yavorska OO, Burgess S (2017). MendelianRandomization: An R package for performing Mendelian randomization analyses using summarized data. Int. J. Epidemiol..

[74] Ip HF (2021). Genetic association study of childhood aggression across raters, instruments, and age. Transl. Psychiatry.

[75] Galán CA, Choe DE, Forbes EE, Shaw DS (2017). Interactions between empathy and resting heart rate in early adolescence predict violent behavior in late adolescence and early adulthood. J. Child Psychol. Psychiatry.

[76] Jennings WG, Piquero AR, Farrington DP (2013). Does resting heart rate at age 18 distinguish general and violent offending up to age 50? Findings from the Cambridge Study in Delinquent Development. J. Crim. Just..

[77] Kavish N, Bergstrøm H, Piquero AR, Farrington DP, Boutwell BB (2020). The longitudinal association between resting heart rate and psychopathic traits from a normative personality perspective. Am. J. Crim. Just..

[78] Sanderson E (2022). Mendelian randomization. Nat. Rev. Methods Primers.

[79] Sanderson E, Richardson TG, Morris TT, Tilling K, Smith GD (2022). Estimation of causal effects of a time-varying exposure at multiple time points through multivariable Mendelian randomization. MedRxiv..

