# Supplementary material for: Resting heart rate and antisocial behaviour: a Mendelian randomisation study
Source: Sci Rep. 2023 Jun 23;13:10212. doi: 10.1038/s41598-023-37123-y (PMC10290077; doi:10.1038/s41598-023-37123-y)
Supplement: Supplementary file 1 — Supplementary Information. [file 41598_2023_37123_MOESM1_ESM.docx]

**Supplementary Material**

**Resting heart rate and antisocial behavior: A Mendelian randomization study.**

Lucy Karwatowska^1*^, Leonard Frach^2^, Tabea Schoeler^3^ Jorim J. Tielbeek^4^, Joseph Murray^5,6^, Eco de Geus^7^, Essi Viding^8^, Jean-Baptiste Pingault^2,9^

Affiliations:

^1^ Great Ormond Street Institute of Child Health, University College London, London, UK

^2^ Department of Clinical, Educational and Health Psychology, University College London, London, UK.

^3^ Department of Computational Biology, University of Lausanne, Switzerland

^4^ Department of Complex Trait Genomics, VU University Amsterdam, the Netherlands

^5^ Postgraduate Program in Epidemiology, Federal University of Pelotas, Pelotas, Brazil

^6^ Human Development and Violence Research Centre, Federal University of Pelotas, Brazil

^7^ Department of Biological Psychology, Amsterdam Public Health Research Institute, Amsterdam, the Netherlands

^8^ Developmental Risk & Resilience Unit, Division of Psychology & Language Sciences, University College London, United Kingdom.

^9^ Social, Genetic, and Developmental Psychiatry, King’s College London, De Crespigny Park, London, UK.

* Corresponding author:

Contact details of the corresponding author:

Lucy Karwatowska

Email: [lucy.karwatowska.18@ucl.ac.uk](mailto:lucy.karwatowska.18@ucl.ac.uk)

Address: Great Ormond Street Institute of Child Health,

30 Guilford Street,

London, WC1N 1EH,

UK

Contents

[eMethods 2](#_Toc134007167)

[LD score regression 2](#_Toc134007168)

[Data sources 2](#_Toc134007169)

[SNP selection 2](#_Toc134007170)

[Supplementary Table S1 STROBE-MR checklist of recommended items to address in reports of Mendelian randomization studies^12^ ^13^ 4](#_Toc134007171)

[Supplementary Table S2 Information on cohorts included in the three genome-wide association studies used in the analyses. 13](#_Toc134007172)

[Supplementary Table S3 Results from the multiple univariable Mendelian randomisation analyses on heart rate variability and antisocial behaviour. 17](#_Toc134007173)

[Supplementary Table S4 Results of the multivariable Mendelian randomisation analysis on resting heart rate and heart rate variability on antisocial behaviour. 19](#_Toc134007174)

[Supplementary Table S5 Results from the positive control analyses using resting heart rate as the exposure and heart rate variability (standard deviation of the normal-to-normal inter beat intervals; SDNN) as the outcome in a univariable Mendelian randomisation analyses. 20](#_Toc134007175)

[Supplementary Table S6 Genetic correlations between heart rate measures and antisocial behaviour. 21](#_Toc134007176)

[Supplementary Table S7 Genetic correlations between resting heart rate and the heart rate variability measures. 22](#_Toc134007177)

[Supplementary Figure S1: A forest plot of the association between independent SNPs for resting heart and antisocial behaviour. 23](#_Toc134007178)

[Supplementary Figure S2: A scatterplot of the association between the SNP effects on resting heart rate and antisocial behaviour. 24](#_Toc134007179)

[Supplementary Figure S3: A funnel plot of the association between the SNP effects on resting heart rate and antisocial behaviour. 25](#_Toc134007180)

# eMethods

## LD score regression

Finally we conducted LD score regression analyses ^4,5^ to estimate genetic correlations between the heart rate measures and ASB. Using *munge_sumstats.py*, we filtered the summary statistics, removing palindromic SNPs, variants with INFO scores < 0.9 and variants which were available for less than 67% of individuals (default parameters).

## Data sources

*Resting Heart Rate*

The summary statistics for resting heart rate were obtained from the largest and most recent GWAS on resting heart rate ^6^. The GWAS included 458,835 individuals from the UK Biobank ^7^ and in addition to the common set of confounders the authors also controlled for age^2^, genotyping array and assessment centre. The original GWAS also controlled for smoking but as smoking is associated with ASB and was not controlled for in the ASB GWAS we asked the authors to rerun the analysis without controlling for smoking. Heart rate was measured during sitting at rest for 2-3 minutes . The reading was taken during blood pressure measurement and/or using the pulse waveform obtained from the finger with an infrared sensor during arterial stiffness measurement. An average was taken where multiple measurements of resting heart rate were available for one individual. The exposure is expressed in beats per minute.

*Resting Heart Rate Variability*

Resting heart rate variability captures the vagal effects on the sinoatrial node co-determining resting heart rate and has also been considered a potential cause of ASB ^8,9^. We obtained summary statistics for resting heart rate variability from the Genetic Variance in Heart Rate Variability (VgHRV) Consortium GWAS ^1^ of 53,174 participants. HRV was measured using three traits: the standard deviation of the normal-to-normal inter beat intervals (SDNN); the root mean square of the successive differences of inter beat intervals (RMSSD); the peak-valley respiratory sinus arrhythmia or high frequency power (pvRSA/HF). All three traits were measured during resting, basal recordings either via electrocardiograms (10-s, 20-s, up to 90 min of sitting or 2 – 12 hour daytime), 24hr Holter monitor, finger photoplethysmograph or Portapres ambulatory heart rate recordings ^1^.

*Antisocial Behaviour*

Summary statistics for ASB were obtained from the Broad Antisocial Behavior Consortium (BroadABC) GWAS, which includes 85,359 individuals from 28 discovery and 5 independent replication samples ^10^. The ASB measures from these samples covered a broad range of behaviours including conduct disorder, aggression and delinquency (see eTable 1 in Supplement for further information on cohort demographics and the types of ASB assessed). The measures were used to derive a single, quantitative measure for ASB. The outcome was a standardised score on a continuous trait scale.

## SNP selection

Prior to the main analyses, we conducted quality control procedures on the GWAS summary statistics. In order for the exposure GWAS to align with the outcome GWAS we retained autosomal SNPs (i.e. SNPs located on non-sex chromosomes) with a minor allele frequency > 0.01 and an imputation information (INFO) score > 0.6. To fulfil the relevance assumption, we used genetic variants that were significantly associated with the exposure (*p* < 5 × 10^−8^). The selected SNPs were then clumped for independence using default parameters from the R package *“TwoSampleMR”* ^11^, excluding any SNPs with a pairwise *r*^2^ > 0.001 within a 10000 kilobase window. The genome-wide significant, independent SNPs for resting heart rate were then harmonised with SNPs from the ASB GWAS, retaining variants that were present in both GWASs. The same clumping and harmonisation parameters that were used for the univariable MR of resting heart rate were used for the heart rate variability data for the multivariable MR.

# Supplementary Table S1 STROBE-MR checklist of recommended items to address in reports of Mendelian randomization studies^12^ ^13^

| **Item No.** | **Section** | **Checklist item** | **Page No.** | **Relevant text from manuscript** |
| --- | --- | --- | --- | --- |
| 1 | **TITLE and ABSTRACT** | Indicate Mendelian randomization (MR) as the study’s design in the title and/or the abstract if that is a main purpose of the study | 1 | Title: “Resting heart rate and antisocial behavior: A Mendelian randomization study.” |
|  | **INTRODUCTION** |  |  |  |
| 2 | **Background** | Explain the scientific background and rationale for the reported study. What is the exposure? Is a potential causal relationship between exposure and outcome plausible? Justify why MR is a helpful method to address the study question | 5 | **Scientific background and rationale:** “Several reviews exist on putative risk factors for ASB, which include environmental and neurobiological factors^14–17^. Physiological markers are particularly important in elucidating potential mechanisms underlying the development of ASB^18,19^”  **Exposure**: “Of these [risk factors], resting heart rate (RHR), defined as the number of heart beats per minute while at rest, is the most well-studied.”  **Causal relationship:** “There have been several meta-analyses conducted on this topic, all suggesting a robust association between these two factors and concluding that RHR is causally related to ASB^20–23^”  **Justification of MR**: “Other genetically informed methods can be used to triangulate these findings by relying on different types of data and assumptions.” |
| 3 | **Objectives** | State specific objectives clearly, including pre-specified causal hypotheses (if any). State that MR is a method that, under specific assumptions, intends to estimate causal effects | 7 | Objectives subsection |
|  | **METHODS** |  |  |  |
| 4 | **Study design and data sources** | Present key elements of the study design early in the article. Consider including a table listing sources of data for all phases of the study. For each data source contributing to the analysis, describe the following: | 7 | Study design subsection |
|  | a) | Setting: Describe the study design and the underlying population, if possible. Describe the setting, locations, and relevant dates, including periods of recruitment, exposure, follow-up, and data collection, when available. | Available in original study |  |
|  | b) | Participants: Give the eligibility criteria, and the sources and methods of selection of participants. Report the sample size, and whether any power or sample size calculations were carried out prior to the main analysis | Available in original study |  |
|  | c) | Describe measurement, quality control and selection of genetic variants | Available in original study |  |
|  | d) | For each exposure, outcome, and other relevant variables, describe methods of assessment and diagnostic criteria for diseases | eTable 1 |  |
|  | e) | Provide details of ethics committee approval and participant informed consent, if relevant | Available in original study |  |
| 5 | **Assumptions** | Explicitly state the three core IV assumptions for the main analysis (relevance, independence and exclusion restriction) as well assumptions for any additional or sensitivity analysis | eMethods | “To evaluate causal effects, the genetic variants must satisfy the following three instrumental variable assumptions: the genetic variants indexing the exposure must be (1) associated with the exposure (relevance); (2) independent of confounders of the exposure-outcome relationship (exchangeability); and (3) only associated with the outcome through the exposure (exclusion restriction)” |
| 6 | **Statistical methods: main analysis** | Describe statistical methods and statistics used |  |  |
|  | a) | Describe how quantitative variables were handled in the analyses (i.e., scale, units, model) | 7 & 8  eMethods | Data sources and measures subsection |
|  | b) | Describe how genetic variants were handled in the analyses and, if applicable, how their weights were selected | eMethods | SNP selection subsection |
|  | c) | Describe the MR estimator (e.g. two-stage least squares, Wald ratio) and related statistics. Detail the included covariates and, in case of two-sample MR, whether the same covariate set was used for adjustment in the two samples | eMethods | SNP selection subsection |
|  | d) | Explain how missing data were addressed | NA | NA |
|  | e) | If applicable, indicate how multiple testing was addressed | NA | NA |
| 7 | **Assessment of assumptions** | Describe any methods or prior knowledge used to assess the assumptions or justify their validity | 9  eMethods | Sensitivity analyses subsection |
| 8 | **Sensitivity analyses and additional analyses** | Describe any sensitivity analyses or additional analyses performed (e.g. comparison of effect estimates from different approaches, independent replication, bias analytic techniques, validation of instruments, simulations) | 9 & 10  eMethods | Sensitivity analyses; Univariable MR Analysis with Heart Rate Variability; Multivariable MR Analysis with Resting Heart Rate and Heart Rate Variability; LD Score Regression subsections |
| 9 | **Software and pre-registration** |  |  |  |
|  | a) | Name statistical software and package(s), including version and settings used | 9 | Statistical analyses subsection |
|  | b) | State whether the study protocol and details were pre-registered (as well as when and where) | NA | NA |
|  | **RESULTS** |  |  |  |
| 10 | **Descriptive data** |  |  |  |
|  | a) | Report the numbers of individuals at each stage of included studies and reasons for exclusion. Consider use of a flow diagram | NA | NA |
|  | b) | Report summary statistics for phenotypic exposure(s), outcome(s), and other relevant variables (e.g. means, SDs, proportions) | NA | NA |
|  | c) | If the data sources include meta-analyses of previous studies, provide the assessments of heterogeneity across these studies | NA | NA |
|  | d) | For two-sample MR:  i.  Provide justification of the similarity of the genetic variant-exposure associations between the exposure and outcome samples  ii.  Provide information on the number of individuals who overlap between the exposure and outcome studies | 7 | Data sources and measures subsection |
| 11 | **Main results** |  |  |  |
|  | a) | Report the associations between genetic variant and exposure, and between genetic variant and outcome, preferably on an interpretable scale | NA | MA |
|  | b) | Report MR estimates of the relationship between exposure and outcome, and the measures of uncertainty from the MR analysis, on an interpretable scale, such as odds ratio or relative risk per SD difference | 9 | Univariable MR analyses with resting heart rate subsection |
|  | c) | If relevant, consider translating estimates of relative risk into absolute risk for a meaningful time period | NA | NA |
|  | d) | Consider plots to visualize results (e.g. forest plot, scatterplot of associations between genetic variants and outcome versus between genetic variants and exposure) | eFigures 1-3 |  |
| 12 | **Assessment of assumptions** |  |  |  |
|  | a) | Report the assessment of the validity of the assumptions | 9 | Sensitivity analyses subsection |
|  | b) | Report any additional statistics (e.g., assessments of heterogeneity across genetic variants, such as *I^2^*, Q statistic or E-value) | 9 | Sensitivity analyses subsection |
| 13 | **Sensitivity analyses and additional analyses** |  |  |  |
|  | a) | Report any sensitivity analyses to assess the robustness of the main results to violations of the assumptions | 9 | Sensitivity analyses subsection |
|  | b) | Report results from other sensitivity analyses or additional analyses | 9 | Sensitivity analyses subsection |
|  | c) | Report any assessment of direction of causal relationship (e.g., bidirectional MR) | 9 | Sensitivity analyses subsection |
|  | d) | When relevant, report and compare with estimates from non-MR analyses | NA | NA |
|  | e) | Consider additional plots to visualize results (e.g., leave-one-out analyses) | NA | NA |
|  | **DISCUSSION** |  |  |  |
| 14 | **Key results** | Summarize key results with reference to study objectives | 11 | First paragraph |
| 15 | **Limitations** | Discuss limitations of the study, taking into account the validity of the IV assumptions, other sources of potential bias, and imprecision. Discuss both direction and magnitude of any potential bias and any efforts to address them | 12 | Strengths and limitations subsection |
| 16 | **Interpretation** |  |  |  |
|  | a) | Meaning: Give a cautious overall interpretation of results in the context of their limitations and in comparison with other studies | 11 | Paragraphs 2-5 |
|  | b) | Mechanism: Discuss underlying biological mechanisms that could drive a potential causal relationship between the investigated exposure and the outcome, and whether the gene-environment equivalence assumption is reasonable. Use causal language carefully, clarifying that IV estimates may provide causal effects only under certain assumptions | NA | NA |
|  | c) | Clinical relevance: Discuss whether the results have clinical or public policy relevance, and to what extent they inform effect sizes of possible interventions | 13 | Conclusions |
| 17 | **Generalizability** | Discuss the generalizability of the study results (a) to other populations, (b) across other exposure periods/timings, and (c) across other levels of exposure | 12 | Strengths and limitations subsection |
|  | **OTHER INFORMATION** |  |  |  |
| 18 | **Funding** | Describe sources of funding and the role of funders in the present study and, if applicable, sources of funding for the databases and original study or studies on which the present study is based | 15 | Funding subsection |
| 19 | **Data and data sharing** | Provide the data used to perform all analyses or report where and how the data can be accessed, and reference these sources in the article. Provide the statistical code needed to reproduce the results in the article, or report whether the code is publicly accessible and if so, where | 15 | Data and data sharing subsection |
| 20 | **Conflicts of Interest** | All authors should declare all potential conflicts of interest | 15 | Conflicts of interest subsection |

This checklist is copyrighted by the Equator Network under the Creative Commons Attribution 3.0 Unported (CC BY 3.0) license.

# Supplementary Table S2 Information on cohorts included in the three genome-wide association studies used in the analyses.

| **Cohort** | **Study design** | **Measures** | **Sample size** |
| --- | --- | --- | --- |
| **Resting Heart Rate** | | | |
| UK Biobank |  | Automated reading during blood pressure measurement; pulse waveform obtained from the finger with an infrared sensor during arterial stiffness measurement. | 458969 |
| **Heart Rate Variability** | | | |
| Atherosclerosis Risk in Communities  Study (ARIC) | Population based | 3-lead ECG; 2 minutes; supine  RMSSD; SDNN; HF | 8262 |
| Cardiovascular Health Study (CHS) | Population based | 24hr Holter monitor  RMSSD; SDNN; HF | 8262 |
| Framingham Heart Study (FHS) | Population based | 2hr ambulatory ECG  RMSSD; SDNN; HF | 1944 |
| FINnish GEnetic STUdy of aRrhythmic Events (FINGESTURE) | Prospective case-control study | 24hr Holter monitor  SDNN; HF | 494 |
| FLEMish study on Environment, Genes and Health Outcomes  – European Project on Genes in Hypertension (FLEMENGHO- EPOGH) | Population based | 12-lead ECG & nasal thermistor for RSA: PSA to estimate HF ranges ; ECG recording for 15 min ; supine  pvRSA | 196 |
| Generation R Study (GenR) | Population based | 3-pole ECG & breathing pattern using a piëzo-electric transducer ; 100-180 seconds ; sitting  HF | 392 |
| Groningen Twin Registry GTR) | Twin study | Type II 3-lead ECGs & respiration with a flexible band around upper thorax; 5 minutes; sitting  RMSSD; SDNN; HF | 134 |
| KOoperative gesundheitsforschung in der Region (KORA S4) | Population based | 2-lead ECG ; 5 minutes ; supine  RMSSD; SDNN; HF | 1617 |
| Multi-Ethnic Study of Atherosclerosis (MESA) | Population based | 12-lead ECG; average from 3 sequential 10-second ECGs; supine; resting  RMSSD; SDNN | 2401 |
| Marine Resiliency Study (MRS) | Population based | Finger photoplethysmograph ; 5 minutes ; sitting  RMSSD; SDNN; HF | 1383 |
| Netherlands Study of Depression and Anxiety (NESDA) | Case-control study | Type II, 3-lead ECG & breathing recorded from thorax impedance ; ~90 minutes ; sitting  RMSSD; SDNN; pvRSA | 1740 |
| Netherlands Twin Register (NTR) | Twin-family study | Type II, 3-lead ECG & breathing recorded from respitrace ; 8 minutes ; sitting  RMSSD; SDNN; pvRSA | 439 |
| Prospective Investigation of the Vasculature in Uppsala Seniors (PIVUS) | Population based | 6-precordial-lead ECG & breathing recorded using custom- made impedance device ; 5-minutes ; supine ; controlled breathing (12 breaths/min)  SDNN; pvRSA | 766 |
| Prevention of Renal and Vascular ENd- stage Disease (PREVEND) | Population based | Beat-to-beat blood pressure pulse wave recording on middle finger (Portapres); 15 minutes; supine  RMSSD; SDNN; HF | 2793 |
| Rotterdam Study (RS1+2) | Population based | 12-lead ECG ; 10 seconds; resting  RMSSD; SDNN | 972 + 985 |
| TRacking Adolescents Individual Lives Survey – CliniCal cohort (TRAILS-CC) | High-risk adolescent cohort | Type II 3-lead ECG; 4 minutes (T1); supine  RMSSD; SDNN; HF | 307 |
| TRacking Adolescents Individual Lives Survey – POPulation cohort (TRAILS-Pop) ^†^ | Population based | type II 3-lead ECG; 4 minutes (T1), 5 minutes (T3); supine  RMSSD; SDNN; HF | 1222 |
| Uppsala Longitudinal Study of Adult Men (ULSAM) | Population based | 6-precordial-lead ECG & breathing recorded using custom- made impedance device from a 24hr recording during normal activity  SDNN; pvRSA | 67 |
| Cardiovascular Risk in Young Finns Study (YFS) | Population based | 2-lead ECG ; 3 minutes ; supine  RMSSD; SDNN; HF | 1827 |
| **Antisocial Behaviour** | | | |
| The National Longitudinal Study of Adolescent to Adult Health (AddHealth) | Longitudinal | Aggression - questionnaire on violent and non-violent activities | 5874 |
| Avon Longitudinal Study of Parents and Children (ALSPAC) | Longitudinal birth cohort | Antisocial behaviour - Edinburgh Study of Youth Transitions and Crime | 2942 |
| Brain Imaging Genetics (BIG) | Population-based | Aggression - Reactive–Proactive Aggression Questionnaire (RPQ) | 862 |
| Collaborative Study on the Genetics of Alcoholism (COGA) | Clinically-based family study | Antisocial personality disorder - Semi-Structured Assessment for the Genetics of Alcoholism (SSAGA) | 7274 |
| CoLaus\|PsyCoLaus | Population-based | Antisocial personality disorder - Semi-structured Diagnostic Interview for Genetic Studies (DIGS) | 4071 |
| Finnish Twin Cohort (FinnTwin) | Population-based | Antisocial personality disorder - Semi-Structured Assessment for the Genetics of Alcoholism (SSAGA) | 2554 |
| The Genetics of Sexuality and Aggression (GSA) | Population-based | Aggression - Buss & Perry Aggression Questionnaire | 2329 |
| Lundbeck Foundation Initiative for Integrative Psychiatric Research (iPSYCH) | Case-control | Antisocial personality disorder – ICD-10 | 24819 |
| Minnesota Center for Twin and Family Research (MCTFR) | Family study | Antisocial personality disorder - Structured Clinical Interview for DSM-IIIR Disorders (SCID) | 5943 |
| Phenomics and Genomics Sample (PAGES) | Population-based | Aggression - Buss-Durkee Hostility Inventory (Assault and Aggression subscales) | 2480 |
| Psychiatric Genomics Consortium (Cardiff sample, CHOP cohort, IMAGE-I & IMAGE-II samples, Barcelona sample, Yale-Penn cohort) | Case-control | Various | 10288 |
| QIMR Berghofer Medical Research Institute (QIMR) | Population-based family study | Aggression - Buss-Durkee Hostility Inventory (Assault and Aggression subscales)  Antisocial personality disorder – questionnaire based on DSM-IV criteria  Arrest – count of number of arrests since 18^th^ birthday | 10363 |
| Spit for Science (S4S) | Population-based | Antisocial behaviour - Semi-Structured Assessment for the Genetics of Alcoholism (SSAGA) | 2187 |
| Twins Early Development Study (TEDS) | Population-based | Antisocial personality disorder – adapted from Edinburgh Study of Youth Transitions and Crime | 3694 |
| Tracking Adolescents’ Individual Lives Survey (TRAILS)^†^ | Population-based | Antisocial behaviour - Antisocial Behavior Questionnaire | 1360 |

*Note. ^†^ Potential overlapping samples.*

# Supplementary Table S3 Results from the multiple univariable Mendelian randomisation analyses on heart rate variability and antisocial behaviour.

| **Method** | ***N_SNPs_*** | ***B*** | ***SE*** | ***p*** |
| --- | --- | --- | --- | --- |
| **RMSSD** |  |  |  |  |
| IVW | 5 | 0.039 | 0.072 | 0.586 |
| MR Egger | 5 | 0.179 | 0.171 | 0.375 |
| Weighted median | 5 | 0.090 | 0.079 | 0.251 |
| MR RAPS | 5 | 0.060 | 0.066 | 0.363 |
| MR PRESSO | 5 | 0.039 | 0.072 | 0.615 |
| Contamination mixture | 5 | 0.100 | 0.119 | 0.146 |
| **SDNN** |  |  |  |  |
| IVW | 5 | –0.005 | 0.132 | 0.971 |
| MR Egger | 5 | 0.606 | 0.484 | 0.299 |
| Weighted median | 5 | 0.097 | 0.129 | 0.454 |
| MR RAPS | 5 | 0.028 | 0.133 | 0.832 |
| MR PRESSO | 5 | –0.005 | 0.132 | 0.973 |
| Contamination mixture | 5 | 0.185 | 0.213 | 0.177 |
| **pvRSA/HF** |  |  |  |  |
| IVW | 4 | 0.029 | 0.045 | 0.522 |
| MR Egger | 4 | 0.105 | 0.091 | 0.367 |
| Weighted median | 4 | 0.047 | 0.040 | 0.244 |
| MR RAPS | 4 | 0.037 | 0.036 | 0.301 |
| MR PRESSO | 4 | 0.029 | 0.045 | 0.568 |
| Contamination mixture | 4 | 0.050 | 0.109 | 0.156 |

*Note. RMSSD = the root mean square of the successive differences of inter beat intervals; SDNN = the standard deviation of the normal-to-normal inter beat intervals; pvRSA/HF = the peak-valley respiratory sinus arrhythmia or high frequency power.*

**Interpretation:**

The results from the univariable MR analyses suggested no causal effect of any of the three measures of heart rate variability on ASB. These results were not affected by heterogeneity as indicated by the IVW and MR Egger estimates. The MR Egger intercept and MR PRESSO estimate suggest no pleiotropic effects and there was no evidence of weak instrument bias (mean *F* statistic range= 57 - 86; all *I^2^_GX_* = 0.99).

MR Steiger filtering showed that the effects on the exposure, heart rate variability, were 61 to 121 times higher than for the outcome, ASB. As the sample size of the underlying GWAS on heart rate variability was relatively small, and only a few significant SNPs were detected, we also repeated the heart rate variability analyses using a more liberal p-value threshold of *p* < 5e-05. The results were comparable with the initial analyses. Of note, although most estimates were not significant, we found a significant effect of HRV (measured by RMSSD) on ASB using the MR Egger estimate (N_SNPs_ = 52, *B* = 0.109, SE = 0.045, *p* = 0.019). However, we also found significant effects of directional pleiotropy using the more liberal threshold for the exposure as indicated by the MR Egger intercept (intercept = –0.005, SE = 0.002, *p* = 0.012) and therefore only report the estimates from the analyses using the more stringent *p*-value threshold of p < 5e-08.

# Supplementary Table S4 Results of the multivariable Mendelian randomisation analysis on resting heart rate and heart rate variability on antisocial behaviour.

| **Method** | ***N_SNPs_*** | ***B*** | ***SE*** | ***p*** |
| --- | --- | --- | --- | --- |
| **Resting heart rate** |  |  |  |  |
| IVW | 18 | 0.016 | 0.145 | 0.914 |
| MR Egger^†^ | 18 | 0.096 | 0.223 | 0.667 |
| **Heart rate variability (RMSSD)** | | | | |
| IVW | 18 | 0.000 | 0.010 | 0.992 |
| MR Egger^†^ | 18 | 0.019 | 0.027 | 0.482 |

*Note*. *RMSSD = the root mean square of the successive differences of inter beat intervals;* ^†^ *estimates when effects are oriented to the respective exposure*.

# Supplementary Table S5 Results from the positive control analyses using resting heart rate as the exposure and heart rate variability (standard deviation of the normal-to-normal inter beat intervals; SDNN) as the outcome in a univariable Mendelian randomisation analyses.

| **Method** | ***N_SNPs_*** | ***B*** | ***SE*** | ***p*** |
| --- | --- | --- | --- | --- |
| IVW | 206 | –0.014 | 0.002 | 6.12 x 10^-17^ |
| MR Egger | 206 | –0.021 | 0.004 | 1.49 x 10^-7^ |
| Weighted median | 206 | –0.016 | 0.003 | 5.64 x 10^-8^ |
| MR RAPS | 206 | –0.014 | 0.002 | 2.62 x 10^-14^ |
| MR PRESSO | 206 | –0.014 | 0.002 | 7.04 x 10^-18^ |
| Contamination mixture | 206 | –0.024 | 0.003 | 8.35 x 10^-11^ |

# Supplementary Table S6 Genetic correlations between heart rate measures and antisocial behaviour.

| **Trait** | ***r_g_*** | ***SE*** | ***Z*** | ***p*** |
| --- | --- | --- | --- | --- |
| Resting heart rate | 0.057 | 0.042 | 1.376 | 0.169 |
| HRV RMSSD | 0.164 | 0.114 | 1.429 | 0.153 |
| HRV SDNN | 0.024 | 0.109 | 0.217 | 0.828 |
| HRV pvRSA/HF | 0.122 | 0.125 | 0.975 | 0.330 |

*Note.* *HRV = heart rate variability,* *RMSSD = the root mean square of the successive differences of inter beat intervals; SDNN = the standard deviation of the normal-to-normal inter beat intervals; pvRSA/HF = the peak-valley respiratory sinus arrhythmia or high frequency power.*

# Supplementary Table S7 Genetic correlations between resting heart rate and the heart rate variability measures.

| **Trait** | ***r_g_*** | ***SE*** | ***Z*** | ***p*** |
| --- | --- | --- | --- | --- |
| HRV RMSSD | -0.5876 | 0.0620 | -9.4820 | 2.49 x 10^-21^ |
| HRV SDNN | -0.5589 | 0.0622 | -8.9832 | 2.63 x 10^-19^ |
| HRV pvRSA/HF | -0.2970 | 0.0566 | -5.2466 | 1.55 x 10^-7^ |

*Note.* *HRV = heart rate variability,* *RMSSD = the root mean square of the successive differences of inter beat intervals; SDNN = the standard deviation of the normal-to-normal inter beat intervals; pvRSA/HF = the peak-valley respiratory sinus arrhythmia or high frequency power.*

# Supplementary Figure S1: A forest plot of the association between independent SNPs for resting heart and antisocial behaviour.

# Supplementary Figure S2: A scatterplot of the association between the SNP effects on resting heart rate and antisocial behaviour.


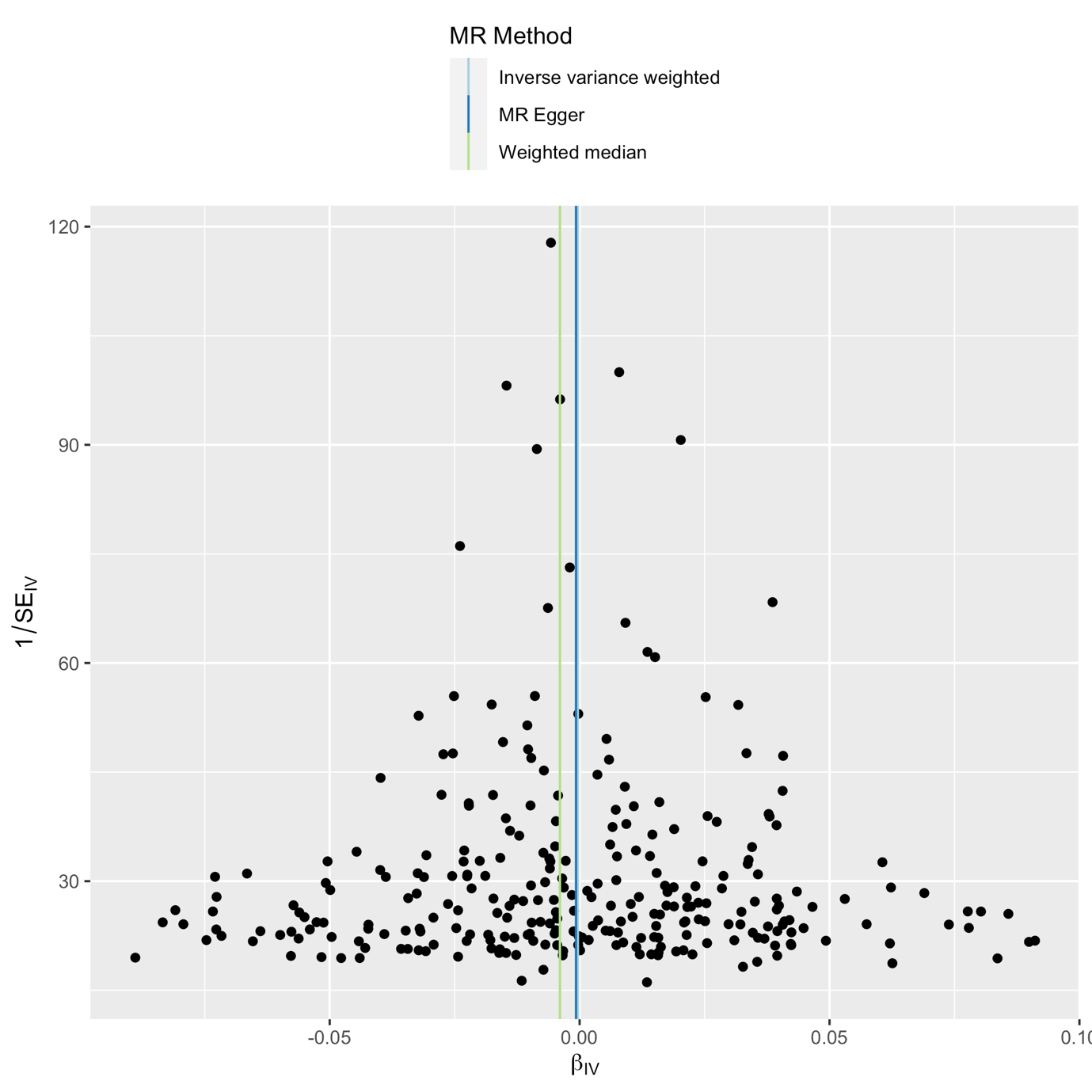


# Supplementary Figure S3: A funnel plot of the association between the SNP effects on resting heart rate and antisocial behaviour.


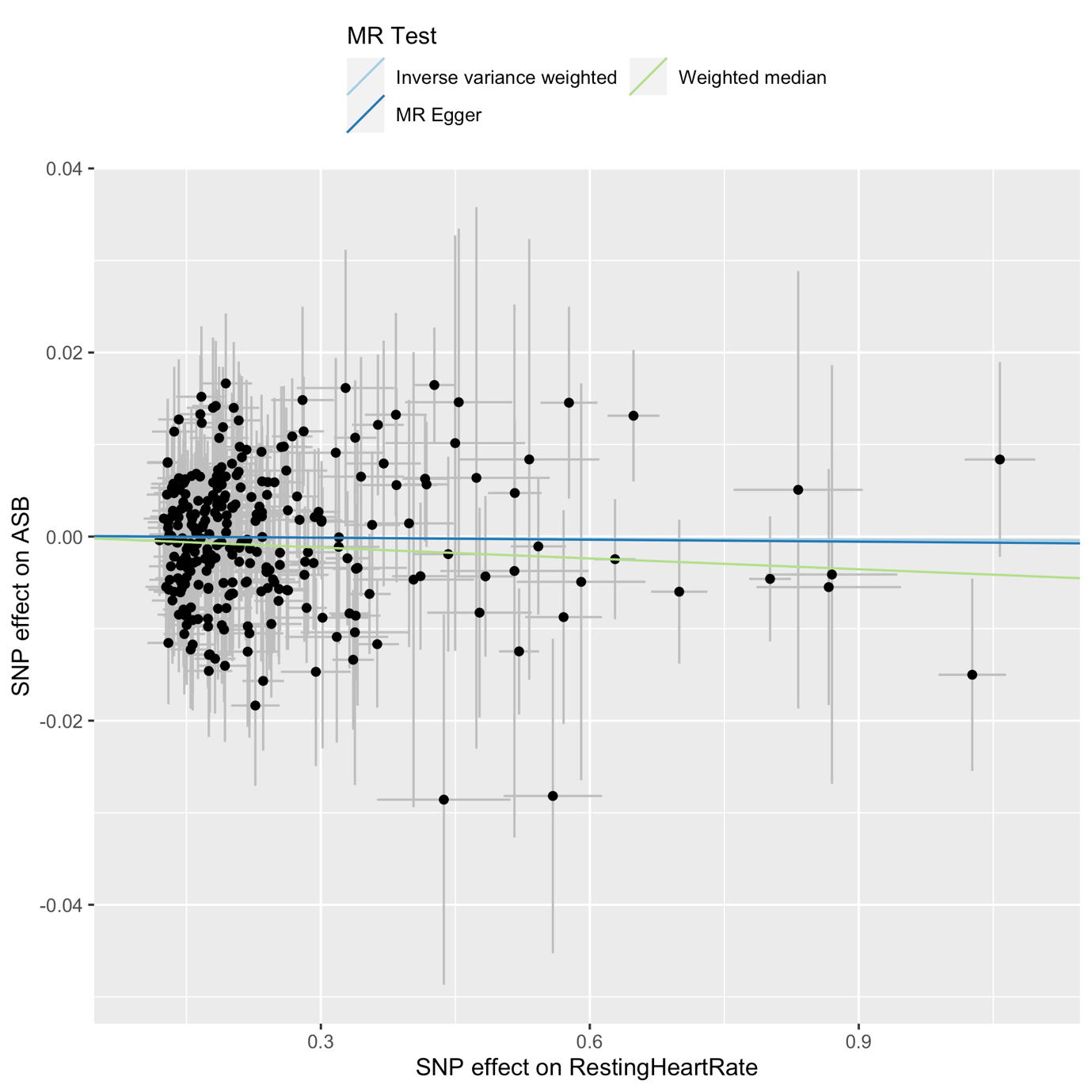


**References**

1. Nolte, I. M. *et al.* Genetic loci associated with heart rate variability and their effects on cardiac disease risk. *Nature Communications* **8**, 15805 (2017).

2. Burgess, S. & Thompson, S. G. Multivariable Mendelian Randomization: The Use of Pleiotropic Genetic Variants to Estimate Causal Effects. *American Journal of Epidemiology* **181**, 251–260 (2015).

3. Rees, J. M. B., Wood, A. M. & Burgess, S. Extending the MR-Egger method for multivariable Mendelian randomization to correct for both measured and unmeasured pleiotropy. *Statistics in Medicine* **36**, 4705–4718 (2017).

4. Bulik-Sullivan, B. *et al.* LD Score regression distinguishes confounding from polygenicity in genome-wide association studies. *Nature Genetics* **47**, 291–295 (2015).

5. Bulik-Sullivan, B. *et al.* An atlas of genetic correlations across human diseases and traits. *Nature Genetics* **47**, 1236–1241 (2015).

6. Zhu, Z. *et al.* Genetic overlap of chronic obstructive pulmonary disease and cardiovascular disease-related traits: A large-scale genome-wide cross-trait analysis. *Respiratory Research* **20**, 1–14 (2019).

7. Allen, N. E., Sudlow, C., Peakman, T. & Collins, R. UK Biobank Data: Come and Get It. *Science Translational Medicine* **6**, (2014).

8. Beauchaine, T. P. *et al.* Respiratory sinus arrhythmia reactivity across empirically based structural dimensions of psychopathology: A meta‐analysis. *Psychophysiology* **56**, e13329 (2019).

9. Beauchaine, T. P. & Thayer, J. F. Heart rate variability as a transdiagnostic biomarker of psychopathology. *International Journal of Psychophysiology* **98**, 338–350 (2015).

10. Tielbeek, J. J. *et al.* Uncovering the Genetic Architecture of Broad Antisocial Behavior through a Genome-Wide Association Study Meta-analysis. *bioRxiv* 2021.10.19.462578 (2021) doi:10.1101/2021.10.19.462578.

11. Hemani, G. *et al.* The MR-Base platform supports systematic causal inference across the human phenome. *eLife* **7**, (2018).

12. Skrivankova, V. W. *et al.* Strengthening the Reporting of Observational Studies in Epidemiology using Mendelian Randomization (STROBE-MR) Statement. *JAMA* under review (2021).

13. Skrivankova, V. W. *et al.* Strengthening the Reporting of Observational Studies in Epidemiology using Mendelian Randomisation (STROBE-MR): Explanation and Elaboration. *BMJ* **375:n2233**, (2021).

14. Derzon, J. H. The correspondence of family features with problem, aggressive, criminal, and violent behavior: a meta-analysis. *Journal of Experimental Criminology* **6**, 263–292 (2010).

15. Hawkins, D. J. *et al.* Predictors of youth violence. *Office of Juvenil Justice and Delinquency Prevention* (2000).

16. Murray, J. & Farrington, D. P. Risk Factors for Conduct Disorder and Delinquency: Key Findings from Longitudinal Studies. *The Canadian Journal of Psychiatry* **55**, 633–642 (2010).

17. Jaffee, S. R., Strait, L. B. & Odgers, C. L. From correlates to causes: Can quasi-experimental studies and statistical innovations bring us closer to identifying the causes of antisocial behavior? *Psychological Bulletin* **138**, 272–295 (2012).

18. Fanti, K. A. Understanding heterogeneity in conduct disorder: A review of psychophysiological studies. *Neuroscience & Biobehavioral Reviews* **91**, 4–20 (2018).

19. Matthys, W., Vanderschuren, L. J. M. J. & Schutter, D. J. L. G. The neurobiology of oppositional defiant disorder and conduct disorder: Altered functioning in three mental domains. *Development and Psychopathology* **25**, 193–207 (2013).

20. de Looff, P. C. *et al.* Heart rate and skin conductance associations with physical aggression, psychopathy, antisocial personality disorder and conduct disorder: An updated meta-analysis. *Neuroscience & Biobehavioral Reviews* **132**, 553–582 (2022).

21. Lorber, M. F. Psychophysiology of Aggression, Psychopathy, and Conduct Problems: A Meta-Analysis. *Psychological Bulletin* **130**, 531–552 (2004).

22. Ortiz, J. & Raine, A. Heart Rate Level and Antisocial Behavior in Children and Adolescents: A Meta-Analysis. *Journal of the American Academy of Child and Adolescent Psychiatry* **43**, 154–162 (2004).

23. Portnoy, J. & Farrington, D. P. Resting heart rate and antisocial behavior: An updated systematic review and meta-analysis. *Aggression and Violent Behavior* **22**, 33–45 (2015).
